# The China-UK Global Health Support Programme: looking for new roles and partnerships in changing times

**DOI:** 10.1186/s41256-020-00156-1

**Published:** 2020-06-03

**Authors:** Lewis Husain, Gerald Bloom, Sam McPherson

**Affiliations:** 1 Institute of Development Studies, Library Road, Brighton, BN1 9RE UK; 2 Itad Ltd, Preece House, Davigdor Road, Hove, East Sussex, BN3 1RE UK

## Abstract

China’s engagement in global affairs has changed substantially in the 2010s. One aspect of the country’s global reorientation has been its increased interest in, and willingness to play a role in, global health. In the early 2010s, the UK Department for International Development (DFID) initiated a collaboration with the Chinese government on a programme to support the country to play a greater and more effective global role in health and explore how the UK and China could work together on issues of key concern and contribute to improved global development outcomes. The programme worked with key Chinese agencies to carry out capacity building, support analysis of China’s approaches to engagement in global health governance and assistance, and provide support to government decision making. It also trialled several small-scale interventions in third countries through which Chinese agencies gained experience of working on health programmes overseas. The article reports on the main findings of an evaluation commissioned by DFID to learn from the programme. The programme provided support at a key time in China’s global reorientation; however, there is a need for continued development of capacity and systems for China to play the role envisaged by the country’s leadership. There is also a need for continued exploration on the part of China and partners of how to effectively collaborate to support improved global outcomes.

## Introduction

The China-UK Global Health Support Programme ran from 2012 to 2019. It supported a wide range of activities intended to support China’s increasing role in global health assistance and governance. The programme was funded by the UK government and was based on the premise that China will play an increasingly important role in global affairs, including global health, that developing Chinese capacity would support more effective Chinese engagement, and that maintaining constructive links between the UK and China would be important following the end of the UK’s bilateral aid relationship with China in 2011. The world faces a range of health challenges that require – and will continue to require – global cooperation, including the ongoing pandemic of COVID-19.

The UK Department for International Development (DFID) commissioned an independent evaluation of the programme, the main aim of which was to learn lessons from this new and innovative partnership with China as its global engagement increased.^1^ This paper draws on findings from the external evaluation and discusses the programme, some key achievements, and what the programme tells us about challenges that lie ahead as the Chinese government, and a range of Chinese agencies, increase their engagement in global health. This is preceded by a discussion of the evaluation methodology in the context of evaluating an experimental programme of this kind. The paper concludes with a discussion of ways forward as China, the UK, and other agencies develop increasingly complex collaborations to respond to current global challenges.

## The GHSP: overview, rationale, and context

The context into which the GHSP was launched was important, and helped create momentum for the programme. This section sketches this important background and provides an outline of the GHSP to situate the discussion in the remainder of the paper.

### The rapidly changing context of the GHSP

The timing of the GHSP and the changes underway in China’s overall engagement increased the significance of the programme – for China, the UK and others. Looking back, it is evident that the GHSP was launched at a transition point, when China’s global engagement was starting to change very rapidly, and fundamentally. This section points to several key trends.

Early in the 2010s, the Chinese leadership started setting out a vision of China’s new global role. Major speeches by Xi Jinping domestically and at major global fora portray China’s increasing engagement as that of a member of a ‘community of shared future for mankind’ (人类命运共同体, also translated as a ‘global community of common destiny’), and underline the importance of China playing a greater, and more active, role in global governance [1]. China’s thirteenth five-year plan, issued in 2015, commits to increasing assistance to other developing countries [2]. These changes point to transformations underway in global governance, and increasing Chinese confidence in its own developmental experience and willingness to engage globally [3, 4]. The most eye-catching initiative of this period is the Belt and Road Initiative, a major, cross-continental initiative aimed at promoting connectivity, industrialisation and development, which was first mooted in 2013 during a visit by Xi Jinping to Kazakhstan.

China has started to put in place institutions to support its changing global role. It has initiated or backed the creation of a number of institutions and financing instruments, including the Asian Infrastructure Investment Bank, the New Development Bank and Silk Road Fund, and made increased financial commitments through existing channels such as the Forum on China-Africa Cooperation (FOCAC).^2^ Major Chinese banks provide large amounts of financing, often in the form of loans, to developing countries – on some metrics this approaches, or has exceeded, US development spending.^3^ In early 2018, the China International Development Cooperation Agency (CIDCA) was launched, to better support and manage China’s overseas cooperation and assistance.

China’s engagement in global health has been deepening and diversifying over the past ten years. FOCAC has consistently included health cooperation, and this has deepened and diversified over time, notably with major announcements in 2015. Health has increasingly been included in China’s cooperation through various south-south groupings or fora, including the BRICS, APEC, the G20, and ASEAN. It also now occupies an important place in the Belt and Road, following the issuing of national strategies for BRI health cooperation [5, 6], and the Belt and Road International Cooperation Forum Roundtable Summit was held in Beijing in August 2017. China’s health assistance has been growing rapidly over the last approximately 15 years, and many discrete initiatives are now underway in a range of countries [7].

### Overview and rationale of the GHSP

It is against the background sketched above that the GHSP was launched. The China-UK Global Health Support Programme (GHSP), was a £12 m partnership between the UK Department for International Development and the Chinese National Health Commission (NHC) and Ministry of Commerce (MOFCOM) that ran from 2012 to early 2019. It was designed as an experiment to explore the new kind of relationship that would be needed following the end of DFID’s bilateral assistance to China.

The programme aimed to support increasing Chinese engagement in global health governance and health assistance, and to explore how the UK and China could work together on issues of key concern to both countries and to contribute to improved global development outcomes. The focus on health responded to a number of things – it built on a history of good relations between DFID and the Chinese health sector, it responded to successes by China in building health systems and improving population health, and it was seen as a comparatively non-contentious area for developing international collaboration.

The design of the GHSP was predicated on the belief that China’s success in improving its own population’s health has lessons for the world that can contribute to improvements elsewhere, and that there is interest in learning from China [8]. Chinese overseas engagement in health is not new, but the GHSP envisaged a diversification of China’s global health engagement, beyond the country’s traditional repertoire, which has principally been composed of building health facilities, dispatching medical teams overseas and donating medical commodities [9].

The GHSP financed activities aimed at building a body of knowledge, analysis and capacity to support China’s increasing engagement in global health. It supported the production of academic papers and policy briefs by Chinese universities and think tanks on the value for other developing countries of China’s experience in building health systems, controlling infectious diseases and in maternal and child health (MCH), as well as on global health governance and approaches to health assistance. It supported several pilots in low income countries (LICs), through which Chinese institutions and local partners carried out interventions that drew on Chinese experience in interventions in Ethiopia, Myanmar and Tanzania, and it supported background analysis of Sierra Leone’s health system to support Chinese involvement in that country. It also established high-level discussion about future bilateral collaboration through the China-UK Global Health Dialogue (Table 1).

**Table 1:** Key GHSP functions at a glance

• Supporting the development of evidence on China’s progress in building health systems and improving population health that could be of relevance to other countries;	
• Supporting analysis of China’s current role in global health governance and health assistance that would help inform the diversification and deepening of China’s engagement;	
• Promoting the development of capacity among Chinese institutions to support China’s global health engagement;	
• Using small-scale pilots to test approaches to overseas programming and employing China’s domestic experience in new contexts;	
• Developing mechanisms to support greater China-UK global health collaboration to support better global development outcomes.	

## Methods

The GHSP was conceived as an experimental programme to help inform new kinds of engagement by China in global health, and new kinds of partnership, including between the UK and China. China’s domestic progress in many areas of development, including improving population health, is globally recognised. The GHSP was predicated on learning from China’s domestic experience in managing changes and developing systems to improve population health, and using this to contribute to developmental outcomes elsewhere. Learning in two main areas was developed through the programme:

- First, China has no template to guide its future global engagement. Its internal development has preoccupied government and researchers for over a generation, and few in government, in research institutions or NGOs, have experience of working in other developing countries or designing or managing development interventions outside China. The country does not yet have mature systems for managing overseas assistance, with the exception of a relatively narrow set of areas, including infrastructure and construction. In health, China’s repertoire of activities is limited.

- Second, other countries and global agencies have no blueprint for building new kinds of partnership with China. The bilateral programmes of most countries and the China programmes of most multilaterals are undergoing substantial change. China’s development, and the increase in Chinese capacity to deal with domestic development issues, reduces the need for outside agencies to support China’s domestic development. Meanwhile, the Chinese leadership states clearly that the country will play a greater and more active role in global affairs, including governance, and development. There is a need to explore new collaborative relationships that will be needed as China becomes increasingly engaged as a development actor outside its own borders.

The external evaluation of the GHSP was commissioned to capture learning in the above areas and inform partnership as China’s engagement increases. It ran alongside the programme, and benefited from long-term engagement with the Chinese implementing agencies, officials in China and the UK, and from discussions with senior officials in global health agencies. It also drew on field visits to countries chosen for GHSP pilots, and on a large amount of information generated by the programme on China’s changing engagement in health assistance and global health governance. It provided a unique opportunity to capture learning from the programme and to inform new partnerships with China as the country’s engagement in global health increases.

Reflecting the experimental nature of the programme, a number of features of the design of the GHSP were deemed to be important for the subsequent evaluation and helped shape the approach:

- The magnitude of the GHSP was modest in the context of a country like China, of continental size.
- Support for the majority of GHSP activity should be primarily considered seed funding, which it was hoped would catalyse more widespread change, for example in capacity development and research.
- The GHSP was not intended to be prescriptive or to push one particular approach in, for example global health engagement or health assistance, but to provide space for exploration of relevant approaches.
- The programme was intended to be reactive, and to be able to capitalise on emerging opportunities, especially for global health engagement, health assistance and China-UK collaboration.
- In many areas, the GHSP did not envisage a linear progression with clearly defined outcomes, but a trend, whose specific outcomes were hard to fully specify in advance.

The evaluation viewed components of the project as ‘probes’ [10, 11] into different aspects of the complex process of ‘capacitating’ actors and institutions (in China, the UK and international organisations) to manage China’s rapidly changing engagement in global health. For each probe we ask about the kind of learning generated, whether it is used and by whom. China’s rapidly increasing role in global development is raising important policy challenges at the highest level in governments and multilateral organisations. In the production of the final evaluation report very senior policy actors in the UK, China and Geneva allocated a substantial amount of time for interviews, indicating the great importance attached to this issue.

## Highlights of findings

The final evaluation built a composite picture of the change in China’s engagement in global health, and the ways that the activities supported by the GHSP have supported this. It focused on several main components. Summary findings, drawing on the final evaluation report, are discussed here (Table 2).

**Table 2:** GHSP – key achievements

• Logframe targets met/exceeded	
• Analyses of China’s domestic experience and its relevance to other countries made widely available	
• Policy analyses contributed to organisational reform for China’s engagement in global health	
• Capacity of Chinese institutions for research and analysis of global health strengthened and China Global Health Network established	
• Pilot projects in Tanzania, Myanmar and Ethiopia had achievements & enabled the government and implementing agencies to gain experience and learn some of the challenges to be overcome for implementation at scale	
• The China-UK Global Health Dialogue established a forum for high level policy discussion	

### The changing context and China’s changing engagement

The evaluation provided an analysis of the rapidly changing context – of how China’s engagement in global health is changing, and the links between this and the overall changes we can see in China’s overseas engagement. The evaluation concluded that China’s engagement in global health was already substantial around the time at which the GHSP was commissioned. Nonetheless, engagement increased over the period of the GHSP and diversified. The GHSP supported changes underway.

China has historically been characterised as principally reliant on bilateral engagement, but it has recently engaged with a variety of fora and institutions. Institutional change in China, including the establishment of the China International Development Cooperation Agency (CIDCA), should accelerate these changes. China’s global health engagement has tended to focus on a few, quite restrictive, kinds of intervention. This appears to be changing, but there is not yet coherence among the new initiatives that are emerging, though certain key themes are consistently emphasised as important. Concrete forms of overseas engagement are diversifying only slowly, though it is possible that this could change, given the high degree of political backing for global health, the changing institutional configuration, and funds increasingly being made available to support cooperation.

### China’s changing partnerships with the UK and global health agencies

The evaluation analysed China’s changing partnerships with the UK and key global health agencies, the ways that aspects of China’s engagement are changing, and the challenges to creating effective partnerships for taking things forward. It concluded that the GHSP helped support dialogue between the UK and China on global health – notably through the China-UK Global Health Dialogue – but noted that partnership between the two countries in global health remains limited.

The evaluation found that there is a general consensus in Beijing, London and Geneva on the potential importance of China’s increasing engagement in global health. There is consensus that China could make important contributions to global efforts to address health needs and strengthen health security. It found that effective partnership in the future will require increased inter-country collaboration (involving regulatory agencies, think tanks, research institutes, consultancy companies, etc) in addition to senior policy makers. This will require an increased investment in effort to establish and build on effective modalities, mechanisms and capacity for collaboration.

### GHSP-supported research and analysis

The evaluation provided an assessment of the research and analysis supported by the GHSP. It found that space for research on China’s global health engagement was important in building awareness and capacity and enabling a process of ‘self-discovery’ of China’s overseas health engagement.

Research supported by the programme contributes to our understanding of what China has done, the limitations of current Chinese approaches to global health engagement, and possible future options. Much policy-focused research has been communicated to policy makers in the health system, though, at the time of the final evaluation, we had not seen major changes in policy reflecting that research. Research supporting the development of a Global Health Strategy supported the adoption of an internal strategy within the NHC, informed government thinking and helped strengthen links across government, though no public strategy had been issued at the time of the final evaluation. Research also provided useful information and insight into elements of China’s domestic health experience and how it may be relevant to other countries, and supported the development of the GHSP pilots.

Informants agreed on the importance of these knowledge activities both as a source of information for the global community and as aids to decision-makers in China. As the scope of China’s engagement grows, the need for a spectrum of knowledge activities to support a learning approach will grow. Collaborations with overseas institutions are likely to play a role in supporting the development of this kind of capacity.

### GHSP-supported pilots

The final evaluation provided an assessment of the implementation of the LIC pilots. The evaluation found that, overall, the pilots demonstrated an ability of Chinese agencies to support overseas programmes in collaboration with in-country partners, and to contribute (to varying degrees) to implementation, though with substantial transaction costs.

The pilots provided an important platform for learning by the programme implementing agencies, and for the Chinese system more broadly, about the complexities of operating overseas and constraints to this. However, the pilots did not substantially engage Chinese and UK agencies in the pilot countries, and engagement with pilot country governments was slow to come. This highlights the limited capacity of both the Chinese and UK systems to cooperate at a country level. Feasible ways to collaborate will require further exploration, and greater support from Beijing and London. The GHSP also showed that adapting ‘experience’ from one country to another is challenging; understanding some of the deeper lessons from China’s experience will likely mean looking more deeply at how China has managed change during its own reforms, rather than just at specific elements of Chinese health policy or practice.

The operation of the pilots, and the learning from them, was conditioned, at least in part, by the way they were managed. Difficulties might have been reduced (and the pilots might have been able to accomplish more) with a different management structure, or more support. Looking forward, China contributing technical assistance to overseas programmes will require greater, and more widespread, capacity to operate in non-Chinese contexts and make Chinese expertise available in ways that are informed by other country contexts.

### China’s developing capacity in global health

The final evaluation provided an assessment of evidence from the GHSP regarding China’s developing capacity in global health, and the contribution of the GHSP. The evaluation found that GHSP provided seed funding to help develop the field of global health in China, which has been important, and valued. The project has built, or strengthened, relationships with core institutions that are likely to be important partners in future collaboration. Training activities were well-received, and helped improve skills, expand networks, and change views of global health and China’s role. They helped motivate many people to work in global health in the future.

Looking forward, there is a need for more opportunities for practical exposure and field experience, and there is a need for stronger systems to support development of this field in China. The programme implementing agencies continue to develop their work in global health, reflecting support from the GHSP, the macro context and perceived openings for institutions with capacity in this area. Substantial government funding was not available at the time of the final evaluation for development of institutional capacity in global health and will need to be developed.

We can see the beginnings of a field in global health in China, but there is a need to provide system-wide support to its development. Saying this, the goalposts have moved – the Chinese government has increasingly high expectations for China’s overseas development engagement, and this will require a broad range of capacity in research and policy support, management and implementation of overseas programmes, and to support engagement in global health governance. Investment will need to be commensurate with the ambitions of the Chinese government. It is not yet clear the extent to which COVID-19 will change the requirements on Chinese agencies to work in global health-related topics, though its impact may be substantial.

Rapid change in the international system, and multiple emerging challenges, will require new kinds of relationships, learning, accommodation and adjustment by all actors – China as well as incumbents. As China’s engagement in global development – including health – deepens, other donors, INGOs, and the multilateral system will also need to develop capacity to collaborate.

## Conclusions and recommendations

The GHSP was intended to have a number of functions, including analysis of China’s experience and options for future engagement, building capacity, trialling new forms of overseas engagement and building dialogue with the UK to support better development outcomes. As discussed above, the programme has contributed to change in all the above areas.

The programme gave Chinese institutions an opportunity to explore China’s potential role in global health, and develop expertise and capacity, at a point at which little support was available from within the Chinese system. It was successful in doing this, though the contribution to change was different in different programme areas. In some cases, the programme demonstrated the challenges of certain approaches to increasing China’s assistance, rather than clear ways forward. However, these are valuable lessons to have learnt from support to a programme at this stage of China’s changing global engagement.

Timing of the GHSP was important, and the programme contributed in a significant way to changes underway in China. The programme was timely, and multiple strands of work it supported contributed to changes underway, as was widely recognised by key stakeholders in China, the UK and Geneva. Obviously, the GHSP was by no means a unique contributor to the evolution we can see in China’s global engagement and, importantly, the contribution that GHSP made would certainly have been less without the transformation in China’s global role with which the programme coincided.

We are entering a period that will be marked by challenges to international cooperation, combined with a need for collaboration for global health. There will be a need for substantial global health initiatives over the short to medium term. Many low- and middle-income countries will experience rapid transformations with big implications for health, including: large development and infrastructure projects stimulating rapid urbanization; climate change, ecological challenges and outbreaks of infectious diseases; demographic change and the growing burden of chronic non-communicable diseases; conflict and civil disturbances leading to displacements of populations and major health problems; and changing patterns of inequality and social exclusion leading to new health challenges. The GHSP highlighted many of the constraints that need to be addressed to enable China to make a substantial contribution in responding to these global challenges.

China’s global health engagement is evolving rapidly, and there is space to develop ambitious collaborations to support it. The transformation underway in China’s global role is leading to substantial increases in the country’s global health engagement and changes in the financing available for developmental initiatives and collaborations. As China’s commitment to an increased global role increases, it will inevitably create expectations on the part of other partners regarding China’s contributions – both technical and financial – as well as tensions. China has its own experience to draw on in its global engagement, and global frameworks and commitments – notably the sustainable development goals (SGDs) and SDG3 – provide a framework to integrate China’s contribution to global debates.

There is agreement by policy-makers in China, the UK and in the WHO regarding the importance of China’s growing engagement in global health and the importance of partnerships. Senior stakeholders in China and the UK consulted during the evaluation agreed that it will be important to implement more ambitious joint programmes to support health system development in low-income countries. The GHSP has provided a way to probe China’s current engagement, China-UK collaboration, and the challenges to moving forward.

The GHSP has provided space to design interventions using elements of China’s domestic experience in new contexts; moving forward, there will be a need for experimentation and rapid learning to develop new and effective models of Chinese health assistance. The GHSP started with the hypothesis that China has experience of managing changes and developing systems that can improve population health. Through the programme, researchers summarised elements of China’s experience to inform the design of overseas pilots. This showed the importance of learning from China’s experience in addressing its own health problems, but that it is limiting to look for simple models of good practice to transfer to other countries. The key lessons from China’s experience appear to be more fundamental, and are linked to *how* the country has managed rapid change. Experimentation supported by the GHSP has shown the potential of a similar approach to change management to inform China’s increasing engagement in global health through trialling small scale interventions and rapidly incorporating learning in new, and more substantial interventions [12].

The GHSP provided space for initial exploration of new forms of Chinese global health engagement, but there is a need for substantially greater exploration and experimentation. China’s researchers and policymakers made substantial use of experimentation, adaptation and rapid learning in guiding the country’s domestic reforms. China’s domestic reform processes have incorporated approaches to summarising and learning from evidence during reform. There is a need for an equivalent approach to guide China’s overseas engagement – incorporating evidence from China and elsewhere, and incorporating these into rapid learning in new contexts.

The GHSP pilots showed the substantial constraints to be overcome by Chinese institutions to enable them to play a leadership role in the management of large health system development programmes. The programme also showed the need to develop widespread system capacity in global health research and analysis, the commissioning, implementation and evaluation of effective overseas interventions, and capacity within government to participate effectively in various global health governance fora. The GHSP contributed in all these areas, but the contribution was, necessarily, limited, given China’s size and the breadth of issues involved. In some areas, such as the development of non-GHSP-supported global health centres and publishing on global health, we can see the beginnings of broader change. However, substantially ‘capacitating the system’ – putting in place the broad system capacity needed to deliver on the role that the Chinese leadership now envisages for the country in global health – will require a major effort and investment, and will take time. Meanwhile, the Chinese Government is exploring the possible role of global/multilateral agencies in managing health system strengthening programmes and other ways to move forward under conditions of limited domestic capacity.

The GHSP helped support dialogue between the UK and China on global health – notably through the China-UK Global Health Dialogue – but partnership between the two countries in global health remains limited. There were only modest efforts to explore on-the-ground partnership in third countries, despite the pilots. Assuming that China’s commitment to global health increases in line with statements from the country’s leadership, there will be a need by international agencies and governments, including the British government, to establish mechanisms to better support coordinated engagement with China, and for capacity to underpin such engagement. There is recognition on all sides of the need to establish new mechanisms, including focal points, to support major increases in activities, joint implementation and increased collaboration. Developing these kinds of collaboration will require learning by all parties to integrate China, its approaches to development and to international engagement into future activities. It will also require learning by Chinese agencies to support the country’s more effective engagement in international and global frameworks. This, in turn, will require investment and leadership on all sides to develop structures and capacity to support deeper and broader collaboration.

## Data Availability

Data sharing is not applicable to this article as no datasets were generated or analysed during the current study.

## Footnotes

1 The evaluation of the China-UK Global Health Support Programme (GHSP) was funded by DFID through the GEFA framework. It was managed by Itad on behalf of the ePact consortium. The opinions expressed are those of the authors and do not necessarily reflect the views of Itad, ePact, or DFID.

2 AIIB: initial capital $100 billion; New Development Bank: subscribed capital base, $50 billion; Silk Road Fund: initial capitalisation $40 billion, subsequently increased.

3 Chinese financial flows do not correspond neatly to development assistance provided by many countries. Only a portion can be classified as official development assistance (ODA), though much may be broadly understood as developmental. The comparison with US spending is based on an analysis provided by AidData (available here).
